# Sequential Treatment by Ozonation and Biodegradation of Pulp and Paper Industry Wastewater to Eliminate Organic Contaminants

**DOI:** 10.3390/toxics12020138

**Published:** 2024-02-08

**Authors:** Jessica Amacosta, Tatyana Poznyak, Sergio Siles, Isaac Chairez

**Affiliations:** 1 Escuela Superior de Ingeniería e Industrias Extractivas, Instituto Politécnico Nacional of México, Ciudad de México 07738, Mexico; amacosta@ipn.mx (J.A.); ssilesa@ipn.mx (S.S.); 2 Institute of Advanced Materials for Sustainable Manufacturing, Tecnologico de Monterrey, Zapopan 45201, Mexico

**Keywords:** pulp and paper, toxic compounds, residual water, combined treatment, ozonation-biodegradation

## Abstract

In this research, the decomposition of toxic organics from pulp and paper mill effluent by the sequential application of ozonation and biodegradation was studied. Ozonation, as a pre-treatment, was executed to transform the initial pollutants into less toxic compounds (such as organic acids of low molecular weights). Biodegradation was executed during three days with acclimated microorganisms that were able to complete the decomposition of the initial organic mixture (raw wastewater) and to achieve a higher degree of mineralization (85–90%). Experiments were performed under three different conditions: (a) only ozonation of the initial contaminants, (b) only biodegradation of residual water without previous treatment by ozone and (c) ozonation followed by biodegradation performed by acclimated microorganisms. In the case of 72 h of biodegradation, the mineralization efficiency reached 85% and 89% after 30 and 60 min of ozonation, respectively. The no significant difference in this parameter coincided with the calculated generalized microorganisms’ consortia specific growing rate $\mu_{max}$ that was reduced from 2.08 × 10^−3^ h^−1^ to 6.05 × 10^−4^ h^−1^ when the ozonation time was longer. The identification of the organics composition by gas chromatography with mass detector (GC-MS) before and after treatments confirmed that the proposed combined process served as a more efficient alternative to secondary and tertiary treatments (mineralization degree between 60 and 80% in average) of the paper industry wastewater.

## 1. Introduction

The principal product of the pulp and paper industry (PPI) is cellulose pulp. This product is derived from the Kraft process applied to wood (cellulose 45–50 wt%, hemicelluloses 20–25 wt%, lignin 20–25 wt% and additional extractable organic compounds 5–25 wt%). The rest of the final elements of the Kraft process are various fibers and recycled material [1]. Cellulose is chemically bleached to remove some organic materials from the pulp. The resulting liquid stream comprises organic chemicals such as lignin and its derivatives: phenols, steroids, tannins terpenes and resin acids and dioxins and their derivative compounds, which are usual components of wood-based materials. These chemicals are removed or converted to chlorinated organic molecules during the bleaching phase of the Kraft process [2]. The major features of pulp and paper mills following the bleaching stage include high color, a very large content of suspended particulates, unregulated levels of chemical oxygen demand (COD) and biological oxygen demand (BOD), plentiful quantities of chlorinated chemicals and an unstable pH range [3,4,5].

These residuals dissolved in PPI wastewater have been considered a serious environmental problem. This is especially concerning because this business is a major buyer of natural raw materials and a significant generator of harmful pollutants (solids, liquids and gaseous effluents). Overall, 72 tons of highly contaminated liquid effluents are generated for every ton of paper produced. This proportion can potentially create irreversible pollution of rivers, lakes, seas and soil [6]. Some plants and animals are poisonous, mutagenic and genotoxic due to all of these substances [7,8]. They do contain lignin and its derivatives. Furthermore, this type of waste effluent frequently contains a complicated mixture of inorganic chemicals that are very recalcitrant and resistant to biological treatment [9,10]. Various sewage treatments for these effluents have been devised over the previous two decades, including physical, biological and chemical approaches or mixtures [11,12,13,14,15,16,17,18,19]. BOD, COD, and total organic carbon (TOC) measurements are widely used to regulate all treatments.

Chemical treatments promote faster decomposition of many different highly concentrated toxic compounds. For example, chemical oxidation by ozone is based on the molecular reaction mechanism or on the indirect reaction aided with the generation of hydroxyl radicals (^•^OH) by the decomposition of gas or with UV radiation [20,21]. N. Kaushalya et al. investigated the ozone-induced discoloration of wastewater samples from the Kraft process [22,23], where the reaction parameters were set to a 240 min reaction time and two different pH values (3 and 10). Under these conditions, there was a substantial decline in phenolic compounds before and after treatment (70% and 52%, respectively). These approaches were effective but can be expensive if mineralization is the main aim of treatment. The sequential application of flocculation precipitation and ozonation (using the flocculation precipitation-like pre-treatment to remove the undissolved organic and inorganic matter by decantation) produces water clarification. With continuous oxidation of dissolved byproducts by ozone after chemical precipitation, the biodegradability was also incremented [20,23].

On the other hand, biological treatment is cheaper and more environmentally friendly, but it requires extended treatment time to eliminate pollutants and exhibits microorganisms’ inhibition when high concentrations of contaminants and/or accumulation of toxic metabolites occur. In the textile and paper industries, different mixtures of microorganisms (bacteria, fungi and algae) have been used to fulfil the biological treatments: *Pseudomonas mendocina*, *Pseudomonas alcaligenes*, *Curvulariainaequalis*, *Phanerochaetechrysosporium*, *Pleurotussajorcaju*, *Pleurotusostreatus* and many others to eliminate organic matter [24,25,26]. Investigators have researched how an *Aspergillus awamori* strain was successful in biodegrading 1 g/dm^3^ of toxic contaminants such as phenol, catechol, 2,4-dichlorophenol and 2,6-dimethoxyphenol and their derivatives. This study confirmed their decomposition after 3, 5, 7 and 8 days of treatment, correspondingly [23,24,27].

Because the wastewater that is created throughout the bleaching cycle contains suspended solids, the primary treatment uses several filters. Secondary and tertiary treatments are typically carried out using conventional methods. Still, in the study, the elimination of soluble organic matter was inefficient because the values of BOD and COD in the treated wastewater sample remained above the reported levels in various local and national legislations [28]. This ineffective result encourages the concept of ozonation followed by biodegradation as a feasible option for treating the complex variety of organic substances prevalent in the pulp and paper industry’s effluent wastewater. This type of treatment technique, comprised of ozonation and biodegradation, was previously evaluated in previous investigations [12,29,30,31].

Decomposition of toxic contaminants was carried out firstly by ozonation to reduce the toxicity of initial organic matter. This fact was justified because ozone promoted the formation of less toxic simple organic mixtures [14,32,33,34]. However, in the mentioned publications, only different model compounds (analytical degree) or actual wastewater were looked at, with no detection of contaminants before and following treatment. Furthermore, the final toxicity of the organic mixture was not determined.

The aim of the present research was to evaluate the effectiveness of organic compounds’ degradation from the real wastewater samples of the bleaching step in the Kraft process by the sequential application of ozonation and then biodegradation. As a secondary objective, adequate operational conditions for both processes were determined in order to reduce the treatment time and to achieve a higher mineralization degree. Additionally, identification of the organic compounds before and after treatment by mass chromatography was performed. This information was used to evaluate the efficiency of the proposed sequential treatment for the elimination of toxic organic contaminants.

## 2. Materials and Methods

All the experiments reported in this section were performed under three different conditions: (a) only ozonation of the initial contaminants, (b) only biodegradation of residual water without previous treatment by ozone and (c) ozonation followed by biodegradation performed by acclimated microorganisms.

### 2.1. Wastewater Samples Preparation

The raw wastewater sample was taken from the bleaching wastewater of a standard Kraft operation. Samples were collected from an unnamed private enterprise in Morelia, Michoacan, Mexico. White liquor generated by sodium hydroxide (NaOH) and sodium sulfide (Na_2_S) was used in the initial phase of this unique Kraft process for cooking wood in digesters at high pressures and temperatures.

The digested wood was removed and cleaned to remove the cooking chemicals and dissolved organics from the fibers. These fibers were then treated in the order listed below. The wastewater treatment consisted of a single cycle of chlorination, followed by two rounds of extraction and dioxin application.

All samples were collected and kept at 4 °C. The aforementioned samples were sterilized in a conventional sterilizer for 20 min at a temperature of 120 °C and a pressure of 0.1 MPa. To prevent the typical foaming of real wastewater and to facilitate sample analysis, the untreated residual water was diluted 1:10 with distilled water and utilized in all tests. Consider that this dilution was not a major issue because the ozone stoichiometric in its reaction with the raw wastewater sample was not affected (therefore, increasing the ozone concentration 10 times solved the effect of dilution).

### 2.2. Ozonation Procedure

**Figure 1:**
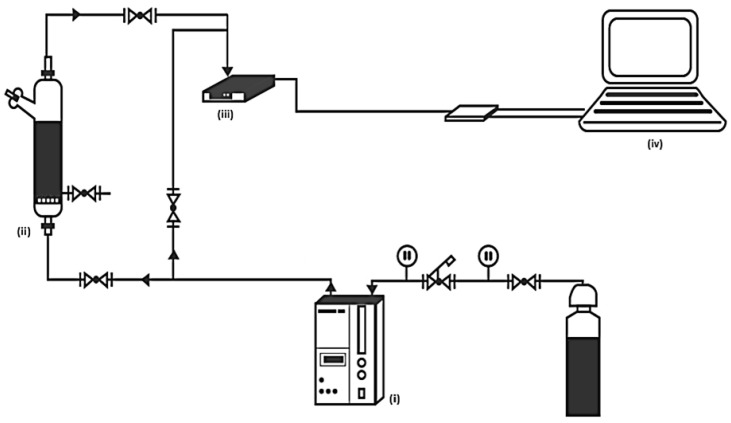
General diagram of ozonation process: (**i**) ozone generator, (**ii**) ozonation reactor, (**iii**) ozone sensor in the gaseous phase, (**iv**) personal computer for capturing ozone concentration.

Ozone was produced from extra dried oxygen with a corona discharge ozone generator HTU500G “Azco” Industries Limited(Langley, Canada) (marked with letter i in Figure 1). The ozone/oxygen mixture flow of 0.5 dm^3^/min with an initial ozone concentration of 10 mg/L was injected through a ceramic porous filter in the semi-batch glass reactor (0.25 dm^3^) with samples of 0.10 dm^3^ (marked with ii in Figure 1) for 30 and 60 min. The Ozone Analyzer BMT 963 “S” (BMT Messtechnik, Berlin, Germany) (marked with iii in Figure 1) provided the ozone concentration determination in the gas phase at the reactor output for the ozone monitoring (via a data acquisition card). A self-designed software based on the Graphical User Interface Development Environment was developed to perform the on-line monitoring task (marked with iv in Figure 1). This software configured and connected the acquisition board and performed the plotting and recording of the ozone concentration.

### 2.3. Biodegradation Procedure

**Figure 2:**
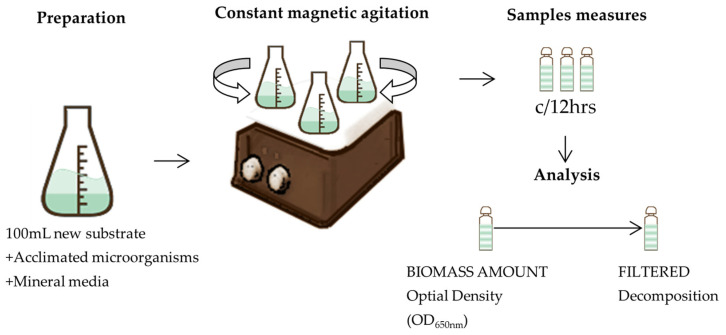
General diagram of biodegradation process.

Operative conditions for the biodegradation stage were fixed using information from a preliminary investigation [35]. The biodegradation stage was performed in a batch reactor with a constant agitation fixed to 300 rpm using a magnetic steering system, with a fixed pH of 7.0 at the ambient temperature (Figure 2). The reaction used supplemented oxygen at a concentration of 21% with a flowrate of 0.5 L min^−1^. Erlenmeyer flasks of 0.25 dm^3^ encompassing 0.1 dm^3^ of mineral culture broth (1.0 dm^3^ of solution contained: 3 g (NH_4_)_2_SO_4_, 0.6 g KH_2_PO_4_, 2.4 g K_2_HPO_4_, 1.5 g MgSO_4_**•**7H_2_O, 0.15 g CaSO_4_ and 0.03 g FeSO_4_) were added with:

- Inoculum of the pre-acclimated consortium (with Ph and A, 1:1—Ph:A) to the sample without previous ozonation.
- Inoculum of the pre-acclimated consortium (with oxalic and formic acids—OFA) to the ozonated samples.

The biodegradation lasted for a period of 168 h. To attain the best possible consortium acclimation conditions, the method of fill-and-draw (F&D) [30] was used. In this study, a complex microbial consortium performed biodegradation that was previously identified [35,36] by extracting DNA from samples using the Easy-DNATM Kit (Invitrogen, Waltham, MA, USA).

The prevalent microorganisms for this consortium are the following bacteria: *Xanthomomna* ssp., *Rhodopseudomona* ssp. and *Ancylobacter* spp. that can degrade chlorohexanes [37], 2-chloroethanol and 2-cloroetylvynyl ether [38], 3-chlorobenzoate [39], etc. During all biological procedures, organic deprivation was determined with respect to time.

### 2.4. Analytical Methods

The color units were determined using the Pt-Co index [28]. The index was created to analyze the pollution levels in wastewater. It is a popular method of comparing the strength of yellow-tinted samples. It is specific to the color yellow and is derived from dilutions of a 500 ppm platinum cobalt solution. The color produced by one milligram of platinum cobalt dissolved in one liter of water is defined as one unit of color on the platinum-cobalt scale. ASTM Designation D1209, “Standard Test Method for Color of Clear Liquids (Platinum-Cobalt Scale)” provides a full description and methodology.

The COD analysis was performed only in the samples with 5 and 60 min of ozonation time by chemical digestion according to method 410.4 (USEPA). The standard COD analysis kit was used with the ready-to-use vials. Low-range Hanna (HI94754A) reagent vials were used to digest the samples. The samples were filtered with a 0.2 µm pore filter after previous digestion for 2 h at 150 °C. After digestion, the samples were measured in the UV-Vis spectrophotometer PerkinElmer Lambda 25 at 600 nm (Waltham, MA, USA).

On the other hand, the TOC analysis was determined by direct injection in the Torch IQOQ equipment (Teledyne-Tekmar, Mason, OH, USA). The solutions were previously filtered by 0.2 µm membrane. These experiments used tert-butanol (TBA) as ^•^OH scavenger with 200 mg L^−1^.

UV-Vis spectroscopy was used to obtain the information of the treatment efficiency and monitoring the organic compound decomposition in ozonation (at 210 nm and 260 nm). These wavelengths were selected because they corresponded to the characteristic maximum absorbance for lignin and its derivatives (260 nm), as well as simple organic acids (210 nm).

The same technique was used to measure the discoloration (at 465 nm) and biodegradation efficiency of the organic source (at 210 nm) of raw wastewater, as well as microbial growth (at 600 nm). The starting compositions of the organic compound mixture (obtained as ozonation process effluent), the byproducts and final products formed in the ozonation procedures, and the biodegradation phases were partly recognized using high-performance liquid chromatography (HPLC, PerkinElmer, USA, Series 200) with UV detector at 210 and 260 nm under different analysis conditions. The Pt-Co scale was determined using this strategy.

In Table 1, the HPLC analysis conditions for the identification of intermediates and final products of the ozonation and biodegradation processes are detailed. The five selected methods of analysis were used to obtain a better separation of the complex mixture contained in the samples of residual water. So, methods I and III were used to obtain the time variation for the concentration of phenolic compounds, and method II was specifically used to obtain the characterization of phenolic compounds and acids. Finally, methods IV and V were specifically used to separate and characterize organic acids’ distribution.

To determine the efficiency of the contaminant’s degradation, the identification of the raw material, as well as the products of the ozonation and biodegradation, was carried out by a gas chromatograph with a mass detector, the GC-MS Claurus600 PerkinElmer, with helium as a carrier gas, an 0.8 cm^3^/min feed flow, split ratio of 1:20 and an analysis temperature of 230 °C. In Table 2, the GC-MS analysis conditions are presented.

A more comprehensive characterization of the original wastewater sample was obtained through successive chloroform (CH_3_Cl) extraction, acid hydrolysis and classification using the Carrez II method. The extraction was performed using the Soxhlet system with a volume of 0.25 L with a liquid volume of 0.1 L. The extraction liquid was CH_3_Cl. The details of this procedure are shown in Table 3.

Three treatment strategies were investigated: ozonation, biodegradation (without prior ozonation treatment or WoPT) and a combination of treatments (a sequential system with ozonation followed by biodegradation). The raw wastewater sample was chosen as a control experiment for comparison purposes.

A set of studies was carried out to evaluate conventional biodegradation in the absence of preceding ozonation. Two distinct ozonation tests were carried out: the first was discontinued after 30 min (M30O) of reaction, and the second after 60 min (M60O). These two precise reaction times were selected because, under the given conditions of the experiment, the byproducts generated in ozonation reached their maximum concentration (measured by HPLC) after 30 min of ozonation but were nearly undetectable (almost degraded) after 60 min. The outcomes of each treatment are detailed below.

A total of three rounds of studies were carried out with the acclimatization technique in mind. The first utilized a sample that had not been pre-treated with ozone, while this acclimated microbe consortium combination was used for comparison (using the WoPT experiments). The second set of studies employed an ozone-treated sample for 30 min (M30O). The third experiment employed an ozone-treated sample for 60 min (M60O). In both cases, the consortium of acclimated microorganisms was dubbed OFA.

The short first-lag phase in microorganism development prevents us from using a standard modeling technique to quantify the effect of acclimation on biomass growth. Furthermore, no specific biomass quantification method was used. As a result, as a suitable technique for characterizing the biomass growth procedure, the biomass accumulation in the 10th cycle of acclimation was modeled as pseudo-monomolecular kinetics [38]. The selected mathematical model was $A=A_{max}\;(1-e^{-bt})$, where *A* is the absorbance measured at the wavelength of 600 nm, $A_{max}$ is its relating maximum absorbance and *t* is the reaction time, while the inverse of $b\;(h^{-1})$ denotes an approximation of the reaction rate.

In regard to the kinetics of the biological process, the specific growing rate (SGR or $\mu_{max}$) was determined using a Monod reaction rate [38].

## 3. Results and Discussion

The primary physicochemical properties of the residual water after dilution were pH = 8, SDT = 3.5 g/dm^3^, color unit of 1400 mg Pt/m^3^, Pt/determined by the Pt-Co technique [28], COD of 670 mg O_2_/dm^3^ and conductivity of 1.0 mS/cm. All of the results were presented for a raw wastewater sample (using the inverse factor of dilution). The diluted sample has been created to be studied using normal spectroscopic and chromatographic procedures. When considering real wastewater, one may notice that this is a common strategy because the levels of color units, as well as concentrations of diluted organics, can be over the maximum level of detectability in the specialist equipment.

An initial GC-MS analysis was made to characterize the organics composition of untreated wastewater. The untreated sample of wastewater contained a wide variety of organic compound groups such as aliphatic, aromatic, chlorinated and polar (acids, esters, ketones, etc.). In general, about 80 organic compounds (C_1_–C_28_) were identified (see Table 3). These organics were chosen from the chromatographs considering the peaks with maximal area.

### 3.1. Ozonation

To determine the ozone consumed in the chemical reaction, the previously proposed parameter named as the saturation constant ($k_{sat}\;[s^{-1}]$) was used to characterize the ozone mass transfer in the ozone–water heterogeneous system. This parameter was calculated according to the method presented in [40,41].

The sample discoloration (obtained at the wavelength of $\lambda_{max}=465\;nm$) was determined along the first 60 min of the ozonation stage. A reduction in color from 140.0 to 30.4 mg Pt/dm^3^ (experimental data are not presented) was obtained (78%).

**Figure 3:**
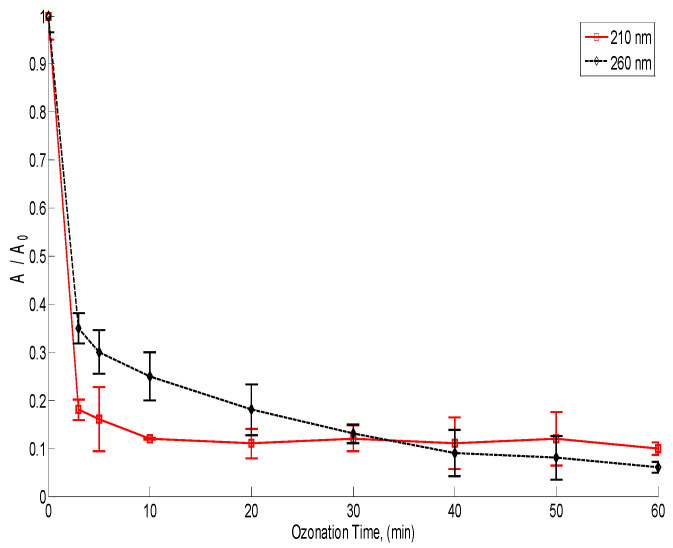
Normalized behavior of absorbance in ozonation during 60 min process. Simple organic acids at 210 nm wavelength and lignin and their derivatives measured at 260 nm wavelength.

In Figure 3, the time variation in lignin as well as its derivatives and simple organic acids in ozonation (obtained by the UV absorbance measured at 260 nm and 210 nm, respectively) are shown. The absorbance of the lignin derivatives was reduced by 67% after the first 3 min of ozonation. On the other hand, the organic acids’ absorbance was reduced by 83% after the first 2 min of ozonation.

On the other hand, the HPLC analysis of intermediates and final products proved the existence of oxalic, fumaric, maleic and formic acids, as well as some phenolic compounds (pseudocumene; 3-methoxy-methyl-ester-propanoic acid; catechol, hydroquinone, dichlorobenzene, decanoic-acid-methyl-ester).

**Figure 4:**
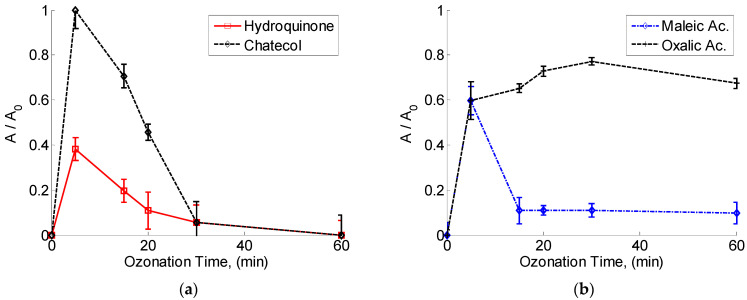
Decomposition behavior of the main organic compounds obtained by HPLC: (**a**) hydroquinone and chatecol, and (**b**) maleic acid and oxalic acid.

The behavior of some recognized hazardous chemicals generated during ozonation is depicted in Figure 4. Catechol and hydroquinone were virtually completely eliminated (99.0%) after 20 and 30 min of ozonation, respectively, because their ozonation reaction constants were larger than 3.0 × 10^5^ dm^3^/(mole s). All other discovered byproducts (except oxalic acid) followed the same dynamics and were degraded during the reaction time.

After 30 min of ozonation, the lignin and its derivatives were almost decomposed with a transformation into simple organic acids (94%). The maleic acid accumulated during the first 5 min and decomposed practically completely after 8 min of ozonation. The oxalic acid, as a recalcitrant compound with a low ozonation constant ($k>40\times 10^{-2}\;{dm}^{3}/(moles)$), accumulated during the 30 min of ozonation from $2.4\;mg/{dm}^{3}$ in the initial residual water sample to $60.0\;mg/{dm}^{3}$ (Figure 4b). This result is in same range of values found by some other authors [42,43,44]. In this particular case, its concentration decreased by 10% during the next 30 min. This behavior can be explained by the effect of OH radicals formed in the case when the reaction is performed at pH > 7 (Figure 4b).

### 3.2. Inoculum Acclimation Prior to Biodegradation

The biodegradation was applied after a preliminary acclimation of microorganisms that was important to improve the mineralization degree of organic matter. This strategy was proposed to make a fair comparison between the results obtained in the ozonation treatment, as well as the sequential process.

**Figure 5:**
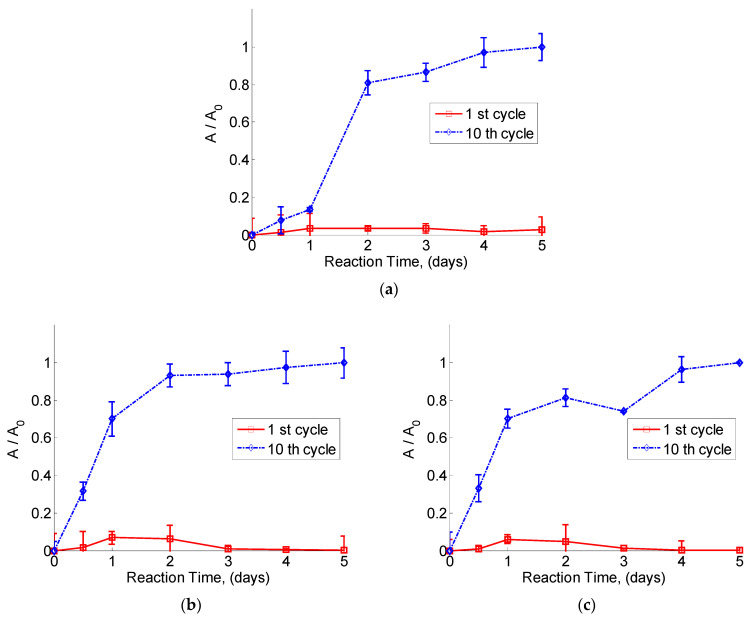
Microorganism’s growth dynamics in the 1st and the 10th cycles of acclimation with the specific organic source: (**a**) without previous ozonation process (WoPT), (**b**) sample previously ozonated by 30 min (M30O) and (**c**) sample previously ozonated by 60 min (M60O).

This stage used a specific carbon source and carried out a series of ten biodegradation phases (5 days each) for consortium maintenance [29]. Because of the amount of culture cycles, the microorganisms were compelled to consume 10 times more substrate during this phase. As a result, their quantity increased, as well as the possibility of increasing the consumption of organic sources. Figure 5a–c describe the growth of microorganisms during the acclimation phase in the three different experiments described above. When the first cycle of 5 days (Figure 5) was evaluated, the biomass growth was 12 times smaller than the one observed when the tenth cycle of acclimation was considered. This condition was obtained with and without the application of a preliminary ozonation process and appeared no matter what the preliminary ozonation time was.

The effect of pre-ozonation was evident on the biomass accumulation. In the case with no previous treatment with ozone, biomass growth was slower than the growth observed in the experiments with pre-ozonation. However, after 10 cycles of acclimation (Figure 5), despite the prior treatment with ozone, the same trend of the biomass accumulation was observed for the sets of experiments when ozonation was used as a pretreatment. The acclimation procedure was efficient to improve the capability of microorganisms to use the specific ozonation byproducts mixture (mainly organic acids) as a carbon source. Moreover, this strategy provided a more adequate form to compare the decomposition efficiency results for the pulp and paper industry. For comparison purposes, the treatment without the acclimation process and without prior ozonation was also investigated. In this case, the constant b was $0.48\;{day}^{-1}$. If the set of microorganisms was acclimated during 10 cycles, this kinetic parameter was modified to $1.13\;{day}^{-1}$. This variation obeyed the selective growing of microorganisms that were able to degrade the compounds found in the water samples. A similar increment of the same parameter was determined for the cases when the pretreatment with ozone was carried out. Similar variations in parameter b were observed when preozonated samples were analyzed.

### 3.3. Biological Treatment without Ozonation

**Figure 6:**
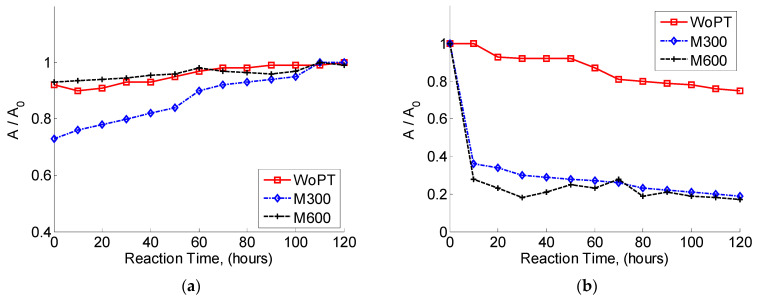
Microbial growth (**a**) and biodegradation of organic matter (**b**) for the WoPT and pre-treated samples of M30O and M60O at the initial pH = 7.

Biodegradation with the acclimated microorganisms showed an incomplete decomposition of the organic compounds considering the complexity and heterogeneity of the wastewater samples’ composition. In general, after 120 h of treatment, the increment of optical density (OD at 600 nm) indicated only partial decomposition of the organic compounds, at 20%:10% in the first 20 min and 10% additional after 50 min of bioprocess, without variation until the end of the experiment. The lines named WoPT in Figure 6a,b demonstrated these facts. These experiments also proved that despite the acclimation procedure, the microorganism’s effectiveness to decompose organic matter was reduced. This fact is confirmed by the total TOC obtained after biodegradation without pre-ozonation.

### 3.4. Combined Treatment: Ozonation before Biodegradation

The total amount of organics’ decomposition obtained as a result of the combined sequential treatment is also shown in Figure 6. In both tests with 30 and 60 min of ozonation, the UV-vis spectrums at $\lambda_{max}=210\;nm$, corresponding to the concentration of simple acids, and the OD at 600 nm were utilized as general markers of the effectiveness of the combined treatment. Following ozonation, most of the organic matter degraded into oxalic acid and other low-molecular-weight acids, which were identified using the HPLC analytical technique.

Following biodegradation, all these organic acids were converted to CO_2_, H_2_O and a few other non-toxic metabolites. In the two treated samples, because of the presence of these chemicals, a sequential treatment based on ozonation, and biodegradation was used. After the first 12 h of the bioprocess, the pollutant level in the two treated samples was reduced by 72% for M30O and 78% for M60O. As can be seen, all organic components began to degrade during the first few minutes of the process. If the organic matter was pre-treated with ozone for 60 min, it was reduced by 78% during the first 30 h, but its concentration did not alter from 30 to 120 h without demonstrating a significant effect of incrementing ozonation time twice. This characteristic can be ascribed to the development of particular metabolites that restrict microbial growth.

At the final stage of the bioprocess (120 h), the total organic matter decrease was 82% for M30O and 83% for M60O, practically without a difference. However, the different ozonation times promoted a significant difference in the biomass dynamics accumulation, which confirms the effect of the initial substrate composition. Notice that the smaller ozonation time (30 min) had the same decomposition effectiveness after 50 h of the combined treatment and maintained this for 120 h. This effect may be used as a partial optimization of this treatment system by reducing the ozonation time. This is regarded as a partial optimization of pulp and paper wastewater treatment. The SGR was estimated without pre-ozonation as $\mu_{max}=8.33\times 10^{-4}\;h^{-1}$. After performing preliminary ozonation for 30 min, the SGR augmented to $2.08\times 10^{-3}\;h^{-1}$. This significant rise in SGR was attributed to the influence of ozone, which reduces the complexity of pollutants while increasing their biodegradability.

The SGR was lowered to $6.05\times 10^{-4}\;h^{-1}$ after 60 min of preparatory ozonation, which can be explained by a reduction in the microorganism’s ability to remove the polar molecules (C_5_–C_17_) generated after this ozonation time. Because of the presence of these polar molecules (organic acids), the SGR was reduced to a level lower than that attained in normal biodegradation. It was established in this example that oxidized products were not appealing to microbes as carbon and energy sources.

The primary refractory product generated during ozonation was oxalic acid, as previously determined by HPLC examination of the treated samples (Table 1). As a result, the dynamics of its decomposition were investigated using the same three sets of experimental circumstances described above (biodegradation, ozonation and the sequential process). The mineralization of oxalic acid was the overall result of biodegradation.

The concentration of oxalic acid was lowered in all three systems, with and without ozonation. The final oxalic acid concentration was reduced by 18% in the non-ozonated sample, while it decreased by 90% in both pretreatment samples. However, when the ozonation period was reduced by half, the best result was obtained when the wastewater sample was ozonated for 30 min (M30O). The mineralization of oxalic acid with other intermediates was validated by measuring TOC, which dropped by 80% after 30 min of ozonation (from 680 to 150 mg C/dm^3^ in diluted water samples).

As can be seen from the results of the GC-MS analysis of the initial untreated water, the water sample had a very complicated composition and contained 70 different organics, including organic acids, fatty acids, esters and ketones, among others, as well as aliphatics and aromatics (Table 3) with a maximal molecular weight of 386. After ozonation, 32 and 40 organics were eliminated from the water samples, depending on the ozonation time (30 and 60 min, correspondently), resulting in degradation efficiencies of 46 and 57% for this step. Moreover, the GC-MS data demonstrated that following ozonation, the water samples comprised different chemical components to the initial sample (Table 4). On the other hand, the treated water samples contained ozonation products which were decomposed in the biodegradation stage (Table 4). As observed, after biodegradation, only 15 compounds remained in the treated samples, which was 21% of the initial composition of organic compounds.

Figure 5 demonstrates the evolution of microorganisms’ biomass considering the presence of the pre-ozonation with different periods. This comparison demonstrates in an integrated manner the effect of ozonating the mixture before biodegradation with different periods. Moreover, the data shown in Table 4 establish the effect on the accumulated byproducts after ozonation. The significant difference in the accumulated profiles of these byproducts justifies the variation in the biomass profiles shown in Figure 5.

The results of the GC-MS analysis results on the original and treated wastewater samples are summarized in Table 5, showing that the compounds were, in general, eliminated. Indeed, after 30 min of ozonation, 23 compounds out of 38 (60%) were removed during the bioprocess. Organic acids, fatty acids, esters and ketones were among the components that were removed. A buildup of heavy alkanes (C_27_, C_28_) and polar molecules was discovered at the end of the tests, which can be described as probable results of bacterial metabolism. Even if there is no precise identification of byproducts generated during successive treatment, a significant variation in organics composition following treatment may occur. Furthermore, there may be a significant decrease in the average molecular weight of the byproducts. This is an indirect confirmation that during biomass growth, a significant amount of organic material with part high-molecular-weight organic compounds are consumed [45,46].

The metabolism of the previously identified microorganisms that form the consortia showed low toxic metabolites because they were low-weight acids that coincide to some preliminary studies [35]. Therefore, the sequential treatment proposed in this study can be considered successful according to these results.

## 4. Conclusions

At the base of the experimental results and discussion, the following conclusions can be suggested:

Following the bleaching phase of the Kraft process, the suggested sequential combination of ozonation and biodegradation of hazardous compounds in pulp and paper mill effluent produced remarkable results. In general, after 30 min of ozonation, a significant quantity of initial harmful pollutants (83%) was reduced, which were subsequently changed into non-toxic organic acids that were easily destroyed at rates of up to 85% after 72 h of biodegradation.

Acclimatization of microorganisms was an important step in the bioprocess treatment since it stimulated the development of microorganisms. After acclimation, the related pseudo-monomolecular constant b associated with biomass growth increased from 0.48 day^−1^ up to 1.13 day^−1^.

Ozonation time played a key role in the enhancement of biomass growth and the decrease in the concentration of toxic organic compounds. In general, without the previous ozonation, the specific growth rate was $8.33\times 10^{-4}\;h^{-1}$ and it increased up to $2.08\times 10^{-3}\;h^{-1}\;$when the sample was ozonated for 30 min. If the preliminary ozonation was carried out for 60 min, this reaction constant was reduced to $6.05\times 10^{-4}\;h^{-1}$.

During experimental settings, a 30 min ozonation period was regarded the best among the evaluated cases since the mineralization degree after 72 h of biodegradation was nearly identical for both preozonated systems (89% vs. 85%) but the ozonation duration was cut in half.

## Figures and Tables

**Table 1:** HPLC analysis conditions for the identification of intermediates and products of ozonation and biodegradation.

**Ozonation**						
	I	II		III		IV
Column	Nova Pack C-18, 250 × 4.6 mm: 10 μm					Prevail Organic Acid, 150 × 4.6 mm
Mobile phase	CH_3_OH-H_2_O (50:50)	C_2_H_3_N-H_2_O (30:70)		C_2_H_3_N-H_2_O (70:30)		H_2_O-KH_2_PO_4_ 25 mmole, pH = 2.5
Flow rate, cm^3^/min	1.0	1.0		1.0		1.0
Injection volume, μL	30	30		30		30
λ, nm	210	260	210	260	210	210
**Biodegradation**						
			V			
Column			Prevail Organic Acid, 150 × 4.6 mm			
Mobile phase			H_2_O-KH_2_PO_4_, 25 mmole, pH = 2.5			
Flow rate, cm^3^/min			1.0			
Injection volume, µL			30			
λ, nm			210			

Numbers I to V refer the selected methods of analysis that we used to obtain a better separation of the complex mixture contained in the samples of residual water. The correspondign details of these methods are described above this table.

**Table 2:** GC-MS analysis conditions.

Column	Elite 5 MS; 30 m × 0.32 mm × 0.25 mm
Mobile phase	Helium
Sample volume, μL	0.5
Split	20%
Flow rate, cm^3^/min	0.8
Injector temperature	230 °C
Temperature program	60 °C after 8 min; 60–240 °C, 30 °C/min; 240 °C after 10 min
Source temperature	230 °C
Transfer temperature	230 °C
Ionization energy, eV	70
Mass, m/z	25 to 400

**Table 3:** Composition of raw wastewater obtained by GC-MS.

Wastewater			Extract by CH3Cl			Acid Hydrolysis H+			Carrez Classification II		
Retention Time	Formula/MW		Retention Time	Formula/MW		Retention Time	Formula/MW		Retention Time	Formula/MW	
1.620	CH_3_OH	32	1.620	CH_3_OH	32	1.62	CH_3_OH	32	1.61	CH_3_OH	32
3.33	C_2_Cl_4_	164	1.72	C_5_H_12_	72	3.29	C_2_Cl_4_	164	4.817	C_5_H_8_O_4_	132
4.633	C_5_H_8_O	84	2.25, 2.37, 2.48	C_5_H_12_O	88	1.83	C_6_H_12_	84	1.782	C_5_H_10_O_2_	102
4.450	C_5_H_10_O_3_	118	5.12	C6H8O	96	2.28	C7H16	100	2.08	C_6_H_12_	84
1.700	C_5_H_12_	72	2.8, 2.92, 3.19, 3.68	C_6_H_10_O	98	6.82	C_12_H_16_	160	5.08	C_6_H_10_O_3_	130
5.620	C_6_H_4_Cl_2_	146	4.95	C_6_H_12_	84	9.49	C_12_H_24_	168	3.066	C_6_H_12_O	100
2.100	C_6_H_6_	78	1.77, 1.8, 1.88, 1.99	C_6_H_14_	86	13.95	C_14_H_12_O_4_	244	6.418	C_6_H_12_O2	116
2.075	C_6_H_10_	82	2.48	C_7_H_16_	100	10.64	C_16_H_22_O_4_	278	4.917	C_6_H_14_O_2_	118
1.98, 2.12	C_6_H_12_	84	3.13, 5.27	C_7_H_14_O	114	11.77	C_18_H_38_	254	1.82, 1.85, 1.87, 1.95	C_6_H_14_	86
2.42, 3.15	C_6_H_10_O	98	2.53	C_7_H_16_O	116				2.28	C_6_H_8_	80
4.650	C_6_H_12_O3	132	8.75, 8.84	C_8_H_12_O_3_	156				4.82, 4.92 5.08	C_7_H_16_	100
1.75, 1.78, 1.8, 1.87, 1.97	C_6_H_14_	86	3.62	C_8_H_18_O	130				4.583	C_7_H_14_O	114
2.849	C_7_H_8_	92	6.32	C_11_H_24_	156				7.085	C_7_H_14_O_2_	130
3.62, 4.25	C_7_H_14_	98	7.001	C_12_H_26_	170				1.948	C_7_H_16_O	116
5.2	C_7_H_14_O_3_	146	9.85	C_13_H_28_	184				7.651	C_8_H_16_O_2_	144
2.15, 2.22, 2.43, 3.45, 4.24, 4.97	C_7_H_16_	100	6.77	C_15_H_32_	212				2.933	C_9_H_20_	128
11.27	C_8_H_14_O_4_	174	9.39	C_15_H_28_O_2_	240				12.57	C_11_H_24_	156
3.660	C_8_H_16_	112	13.28, 14.02	C_20_H_42_	282				15.27	C_18_H_38_	254
2.50, 2.58, 2.75, 2.8, 2.88, 3.2	C_8_H_18_	114	18.67	C_26_H_54_	366				13.64	C_20_H_42_	282
4.68, 5, 5.08, 5.17, 5.25, 5.42, 5.67	C_9_H_12_	120									
4.967	C_9_H_16_O	140									
4.57, 4.77,	C_9_H_18_O	142									
4.38, 6.751, 6.98	C_9_H_20_	128									
6.98, 8.22	C_10_H_8_	128									
5.317	C_10_H_20_	140									
4.833, 6.30, 8.068	C_10_H_22_	142									
5.37, 6.15, 6.20, 6.45, 6.70,	C_10_H_24_	144									
7.65, 7.35, 7.75	C_11_H_10_	142									
7.070	C_11_H_16_	148									
8.068	C_12_H_10_	154									
5.7, 5.8, 6.02, 6.07, 7.07	C_11_H_24_	156									
8.22, 8.29, 8.3, 8.4, 8.47	C_12_H_12_	156									
8.59, 8.64	C_13_H_12_	168									
7.55, 11.29	C_12_H_26_	170									
8.69, 8.80–8.97	C_13_H_14_	170									
9.60	C_14_H_12_	180									
9.09, 9.23	C_14_H_14_	182									
9.42	C_14_H_16_	184									
10.62	C_14_H_18_O_4_	250									
16.94	C_16_H_22_O_4_	278									
10.42	C_17_H_34_O_2_	270									
9.84	C_18_H_38_	254									
11.47	C_19_H_36_O_2_	296									
14.52, 16.24	C_26_H_54_	366									
16.51	C_28_H_34_O	286									

**Table 4:** Composition of wastewater after ozonation and after biodegradation. RT stands for retention time.

No	Compounds	MW	M30O		M60O	
			RT before Biodegradation	RT after Biodegradation	RT before Biodegradation	RT after Biodegradation
1	CH_4_O	32	1.30	1.32	1.30	1.27
2	C_5_H_10_O_3_	118	4.47	5.68	4.47	
3	C_6_H_6_	78	2.09			
4	C_6_H_10_O	98		3.19		
5	C_6_H_12_O	100		2.58		2.58
6	C_6_H_12_	84		1.98		
				2.13		
7	C_6_H_14_	86		1.87		
8	C_6_H_12_O_2_	116		3.37		
9	C_6_H_12_O_3_	132		3.98	4.67	3.98
10	C_7_H_8_	91	2.82			
11	C_7_H_14_	98	2.03			
			2.25			
12	C_7_H_12_O_4_	160			4.78	5.23
13	C_7_H_14_O_2_	130			2.13	
14	C_7_H_14_O_3_	146		5.28		
15	C_7_H_16_	100	2.12			2.87
			2.28			
			2.50			
16	C_8_H_10_	106	3.97		3.97	
			4.07			
			4.33			
17	C_8_H_18_	114	2.22		4.33	
			2.43		2.43	
			2.52			
			2.90			
			3.32			3.37
			6.42			
19	C_8_H_14_O	126			2.52	
20	C_9_H_12_	120	5.01	4.68	4.40	4.68
			5.08		5.00	
			5.27		5.08	
			5.45		5.42	
					5.45	
21	C_9_H_18_O	142			4.78	
22	C_9_H_20_		4.40		2.89	
23		134	5.68	5.40	5.70	5.40
			6.48		5.98	
					6.48	
24	C_10_H_18_O	154			4.88	
25	C_10_H_22_	142	5.46			
26	C_11_H_10_	142	7.27		7.65	
27	C_11_H_16_	148	6.68		6.63	
28	C_11_H_24_	156	6.30		6.30	
29	C_12_H_10_	154	8.07		8.07	
30	C_12_H_12_	168	8.38		8.40	
31	C_13_H_14_	170	8.97		8.95	
32	C_13_H_28_	184	7.55		11.27	
33	C_14_H_10_	178				10.09
34	C_14_H_30_	198	6.98		6.98	
35	C_14_H_18_O_4_	250			10.64	
36	C_17_H_34_O_2_	270	5.80		10.42	
37	C_27_H_56_	380	9.11			9.13
			9.22			10.40
			10.42			14.44
			11.27			
38	C_28_H_58_	394		15.01		15.72
				16.29		16.41
				18.58		18.59
Total number of compounds			38	15	30	15

**Table 5:** Summary of the results obtained by the GC-MS technique of the raw and treated wastewater samples (Compound content, wt%).

Compound	Original Wastewater	After Ozonation M30O	After Biodegradation	After Ozonation M60O	After Biodegradation
Chlorinated Hydrocarbons	4.4	-	-	-	-
Alkanes	17,7 (C_5_–C_12_, C_18_, C_26_)	47.4 (C_7_–C_14_, C_28_)	53.3 (C_28_)	13.3 (C_27_, C_28_)	53.3 (C_27_, C_28_)
Unsaturated Hydrocarbons	33.3	50.0 (C_10_–C_14_)	-	53.4 (C_11_–C_14_)	20.0
Polar Hydrocarbons	31.1 (C_5_–C_28_)	-	46.7 (C_5_–C_6_)	33.3 (C_5_–C_17_)	26.7
Aromatic Hydrocarbons	13.5	2.6	-	-	-
Total	100	100	100	100	100

## Data Availability

All data used or obtained during the development of this study may be available under appropriate request to the Corresponding Authors.
